# Low lung function in the developing world is analogous to stunting: a review of the evidence

**DOI:** 10.12688/wellcomeopenres.15929.2

**Published:** 2020-11-18

**Authors:** Navya Mishra, Sundeep Salvi, Tanica Lyngdoh, Anurag Agrawal

**Affiliations:** 1 Public Health Foundation of India, Delhi, India; 2 Academy of Scientific and Innovative Research, Ghaziabad, India; 3 Chest Research Foundation, Pune, India; 4 CSIR Institute of Genomics and Integrative Biology, Delhi, Delhi, India

**Keywords:** Stunting, malnutrition, low lung function, restrictive lung function, interventions for low lung function, population health, developing nations

## Abstract

**Background: **Low vital capacity, one of the consequences of restricted lung growth, is a strong predictor of cardiovascular mortality. Vital capacity is lower in the developing world than the developed world, even after adjusting for height, weight and gender. This difference is typically dismissed as ethnic variation, adjusted for by redefining normal. Whether this is a consequence of stunted lung growth, rather than just genetically smaller lungs, has not been investigated in detail. Therefore, we sought to compare factors implicated in both stunting and lung development, particularly in the developing world.

**Methods: **We conducted a manual screen of articles identified through Google Scholar and assessed risk of bias. No language restrictions were applied, so long as there was an associated English abstract. We queried VizHub (Global Burden of Disease Visualization Tool) and Google Dataset search engines for disease burden and genome wide association studies.  The scope of the article and the heterogeneity of the outcome measures reported required a narrative review of available evidence. To the extent possible, the review follows PRISMA reporting guidelines.

**Results:** Early life influences operate in synergism with genetic, environmental and nutritional factors to influence lung growth and development in children.  Low lung function and stunting have common anthropometric, environmental and nutritional correlates originating during early development. Similar anthropometric correlates shared chronic inflammatory pathways, indicated that the two conditions were analogous.

**Conclusion:** The analogy between poor lung function and stunting is conspicuous in the developing world, with malnutrition at the center of non -achievement of growth potential, susceptibility to infectious diseases and intrauterine programming for metabolic syndrome. This counter the idea of redefining the normal for lung function measurements, since observed inter-ethnic variations are likely a mix of natural genetic differences as well as differences in nurture such that reduced lung function reflects early life adversities.

## Introduction

Forced Vital Capacity normalized to height was found to be an independent indicator of cardiovascular risk in the Framingham Heart Study Cohort. A series of landmark studies since then have cemented the role of spirometry as a prognostic tool for non-communicable disease outcomes in general and cardiovascular disease outcomes in particular ^1– 6^. This has allowed lung function to transcend its status as an indicator of respiratory disease severity to a predictor of all-cause and cause specific mortality.

While the development of ethnic and geographical reference equations for lung volumes may incorporate differences in body habitus, the validity of these equations would rely heavily on assumptions regarding the reference population being ‘healthy’ ^7^. Adverse socio-economic and environmental factors prevalent in the developing world obscure the definition of a phenotypically ‘healthy’ population. Here, a non-invasive indicator such as lung function, influenced by perinatal growth conditions, growth faltering, repeated infections and malnutrition resulting in a chronic inflammatory state, reliably reflects health across the life course.

Defined as height-for-age more than two standard deviations below the WHO Child Growth Standards Median ^8^, stunting is an equally powerful proxy for similar exposures encountered early in the life course. As both lung function and early growth are strongly associated with and defined by linear growth, influenced by similar perinatal factors, and culminate in an elevated risk of non-communicable diseases sharing chronic inflammatory origins, the associations and intersections between poor lung function and stunting could reveal a roadmap for common interventions for both conditions. In this case, the idea of both stunting (as an outcome of growth faltering conditions) and poor lung function were examined as analogous processes, based on the hypothesis that both conditions appeared to be steeped in similar origins, had similar intermediate indicators and culminated in an elevated risk of similar outcomes.

The intersecting pathways culminating in growth faltering and poor lung function signal towards the pathological origins of poor lung function. Assumptions regarding the reference population being healthy are central to the generation of ‘normal’ spirometry values for a given population. These assumptions are violated in populations experiencing adverse developmental conditions leading to growth faltering and undiagnosed asymptomatic cardiometabolic disease. Therefore, this analogy merits detailed investigation.

## Methods

We searched through Google Scholar and PubMed between June 2019-December 2019, with the last search performed on December 29 ^th^ 2019. The search was conducted in two main phases. A primary search was conducted to identify the main risk factors implicated in stunting, growth faltering and reduced lung function, using keywords such as “stunting”, “lung function”, ”lung capacity”, ”forced expiratory volume”, ”forced vital capacity”, “lung development” and “growth faltering”. We also conducted a brief analysis of GBD 2017 data to describe the role of socioeconomic influences affecting both conditions.

We identified relevant articles using the search terms grouped according to risk factors, such as maternal nutrition and anthropometry, nutrition, anthropometry, environmental factors, sanitation, genetic and epigenetic factors. 

### Eligibility criteria for studies

Randomized controlled trials, cohort studies, case-control studies and cross-sectional studies in which participants were aged 0–25 years were included. In addition, the following inclusion criteria were used:

•    English abstract available

•    Human studies

•    Participants of studies are children aged 0–25

•    Published after 1990

•    Papers contained original data and were full-length peer-reviewed

### Outcomes of interest

The following outcomes of interest were included and assessed:

•    Lung function as measured by spirometry (e.g. FEV1, FVC, PEF, FEF 25–75 and FEV1/FVC).

•    Airway resistance as measured by R5, X5, R20, X20 and similar derivatives

•    Respiratory infections

•    Respiratory mortality

•    Chronic obstructive pulmonary disease (COPD)

•    Stunting

•    Related comorbidities

### Synthesis of results

Studies meeting the inclusion criteria were grouped according to the risk factors identified during the primary search. Risk factors included intrauterine growth restriction, malnutrition, sanitation, air pollution, cigarette smoking, genetic and epigenetic factors. Subsequent searches were then performed to examine the relationship between each set of risk factors with both stunting and lung function.

Evidence from community-based studies and mechanistic studies were then examined together to identify the interrelationships between stunting and lung function and organized into sections according to chief risk factors implicated in both conditions. Consequently, the results were summarized in a narrative form. The heterogeneity in outcomes of the studies included in the review necessitated a narrative review, and to the extent possible, this review follows PRISMA guidelines. 

## Results

### Higher burden of stunting and low lung function in the developing world indicates the role of socioeconomic influences

Evidence of reduced lung function in the developing world emerged from the Prospective Rural Urban Epidemiological Study (PURE), which investigated global variation in lung function in healthy populations by region. Compared with North American or Europe, FEV1 adjusted for age, height and sex, was 31.3% lower in south Asia, 24.2% lower in Southeast Asia, 12.8% lower in East Asia, 20.9% lower in Sub-Saharan Africa, 5.7% lower in South America, and 11.2% lower in the Middle East. Similar and larger differences existed for FVC ^9^.

While it is conceivable that low lung function in apparently healthy communities in developing nations represents a healthy but genetically smaller lung than western populations, this should not be assumed given the high rates of chronic respiratory and cardiovascular disease mortality.

The prevalence of reduced FVC was strongly associated with education level and biomass index in a study assessing the prevalence of reduced FVC and associated risk factors in the African population ^10^. In the Burden of Lung Disease study, the prevalence of a restrictive spirometry varied widely by site and gender, affecting as few as 4.2% of males in Sydney to as many as 48.7% of females in Manila ^11^.

Indeed, across Burden of Obstructive Lung Disease (BOLD) study sites, with NHANES III as the reference data, Restrictive Lung Function (RLF), defined as Forced Vital Capacity <80% of predicted, was extremely common in the developing world, but less so in affluent nations ^12, 13^. The use of reference equations that are generated primarily in order to account for genetic heterogeneity, for developing world populations experiencing large variations in developmental conditions, could obscure the true prevalence of reduced lung function. Epigenetic mechanisms that may persist for generations make it even more difficult to fully exclude environmental effects on development.

Stunting was estimated to affect 21% of children under 5 years of age globally, more than half of whom live in Asia and a third in Africa. It is distinguished from other nutritional disorders that affect the life course, in that it is irreversible if not addressed within the first 1000 days. Even if catch-up growth does occur, it predisposes the individual to an elevated risk of metabolic dysfunction in later life ^14^. The human capital loss resulting from stunting is likely to overwhelm health systems of developing nations, ill-equipped to rehabilitate the 39.6 and 96.8 million affected children in low-income and low-middle-income countries, respectively. In comparison, 2.1 million children are affected in high-income countries. This is because indicators of stunting such as low per capita income, household food insecurity, repeated infections due to substandard sanitation and unsafe water, and poor maternal health and birth outcomes, follow a socio economic dependent pattern ^15^. Here stunting differs from restrictive lung growth in that except for its contribution to a long term chronic inflammatory state, it’s effects may be reversible to an extent if addressed during the first 1000 days of life, whereas the consequences of restrictive lung growth persist throughout the life course ^16^.

An analysis of the GBD 2017 data, showed a relationship between Socio-Demographic Index (a development metric used by the Global Burden of Disease Study), and deaths due to respiratory tract infections, stunting, wasting, preterm birth, diarrhea, suggesting that both disease, and risk factors implicated in both conditions followed a socio economic dependent pattern (see *Extended data*) ^15^.

As a sequel to intrauterine growth restriction (IUGR), stunting appears to mirror the pathology of restrictive lung function. When accompanied by rapid weight gain during infancy and later childhood it is an intermediate predictor of risk of metabolic dysfunction during adulthood. Early manifestations such as low birth weight, short stature and a high Cormic index (upper to lower body segment ratio) too are common to restrictive lung function and stunting. Once the foundation of altered metabolic programming is laid during the perinatal period, continued exposure to adverse environmental conditions result in a heightened inflammatory state and predispose individuals to a high risk of chronic metabolic diseases ^17^.

This exacerbation of disrupted metabolic programming during development has been a major mechanism influencing epidemiological transitions in the developing world, where populations traditionally experiencing high rates of IUGR, malnutrition and stunting are now confronted with an increasing prevalence of chronic metabolic diseases. It is important to note that nations in different stages of epidemiological transition would exert opposing effects on the association between SDI and deaths due to chronic diseases.

This is observed in the moderately high, positive correlation between SDI and Ischemic Heart Disease (GBD 2017). This association would likely grow and stabilize in future, as most nations would tend to a lower epidemiological transition level (ETL). The interplay between socioeconomic status and variations in ETL across populations/communities needs to guide the design of interventions for both stunting and restrictive lung function.

### Examining the mediators of stunting and low lung function: interplay of ethnicity, environment and access

In the arid regions of rural Tanzania, stunting mediated growth retardation was associated with cultivated land size, gender and age of the child, duration of breastfeeding, household size, use of iodized salt, the distance to a water source, literacy status and BMI of the mother ^18^. Stunting influenced deviations from predicted lung function values among 208 stunted and 365 non-stunted children in Tibet. These differences were compatible with the effects of retarded growth and lung maturation characteristic among stunted children ^19^.

Similarly, a Peruvian study with data from 553 asthmatic children, reported an association between food insecurity and poorer Asthma control ^20^. Asian children in low SES environments, with indications of stunting, such as short stature and low BMI, had the highest FEV _1_/FVC ratio on average. This is because exposures implicated in stunting result in reduced lung growth and low FVC values, and due to their limited and indirect effect on FEV _1_, result in a high FEV _1_/FVC ratio. Therefore, stunting manifests with restrictive lung function, as low values of FVC with normal to high FEV _1_/FVC ratio ^21^.

One point of discord in the analogy between growth faltering and poor lung function is the varying extent to which both conditions could be governed by genetic factors. 

A study examining differences in lung function between Asian and White school-children ages 6-11 in Leicester ^22^, found differences in ethnicity to be significantly associated with lung function, after adjusting for socio-economic factors (which additionally determine access to nutrition and exposure to air pollution). While ethnic differences were seen to exist, these differences could not be attributed to clear anthropometric correlates. This was also observed in a study examining the differences in lung function in 112 young adults, where differences in anthropometric indicators did not explain ethnic differences in lung function ^23^. In the CARDIA cohort too, race and sex differences in lung function appeared to persist despite detailed adjustment for frame size ^24^. The mechanistic influences of ethnicity (‘nature’) are speculated to manifest through surrogate markers for height, such as proportions of leg length to body height, sitting height, or differences in inspiratory muscle strength or lung compliance ^2^. It is also plausible to assume that the difficulty in untangling the effects of genetic and environmental influences could be attributable to the low-penetrance nature of genes which regulate lung development and function. The nature and extent of gene-environment interactions are intrinsic to the mode of action of many common, low-penetrance genes ^25^. 

These in turn may work in tandem with developmental mediators such as the intra uterine growth environment and early life exposures. Part of the residual variability could be attributed to unmeasured determinants, which are likely to be both genetic and epigenetic. It must however be noted that ethnicity is a complex imperfect socio-political construct and can be a poor surrogate of genetics, as argued elegantly by Quanjer *et al.* ^26^. 

In this context, while attained lung function may be considered a phenotypic expression of both genetic endowment and childhood environment, stunting primarily appears to be a consequence of deprivation and inequality encountered during development. Disruptions in environmental factors such as maternal nutritional status, feeding practices, hygiene and sanitation, frequency of infections and access to healthcare are the major determinants of the risk of stunting ^27^. 

IUGR is an interesting intersection point for the purpose of this study that is environment driven, highly prevalent in developing nations, results in smaller organs and low birth weight infants with a higher susceptibility to diarrheal and lower respiratory tract infections. This sequence, which may even persist beyond a single generation since maternal size is in itself a limiting point for fetal growth, leads to repeated growth faltering and reinfections, which are implicated in stunting ^28– 31^. IUGR, as a consequence of maternal loss of growth potential, may be considered a point of convergence in the pathways for poor lung development and stunting, for which we have more data than either of the two alone. 

### IUGR: The cornerstone for stunting and low lung function

**Figure 1.**
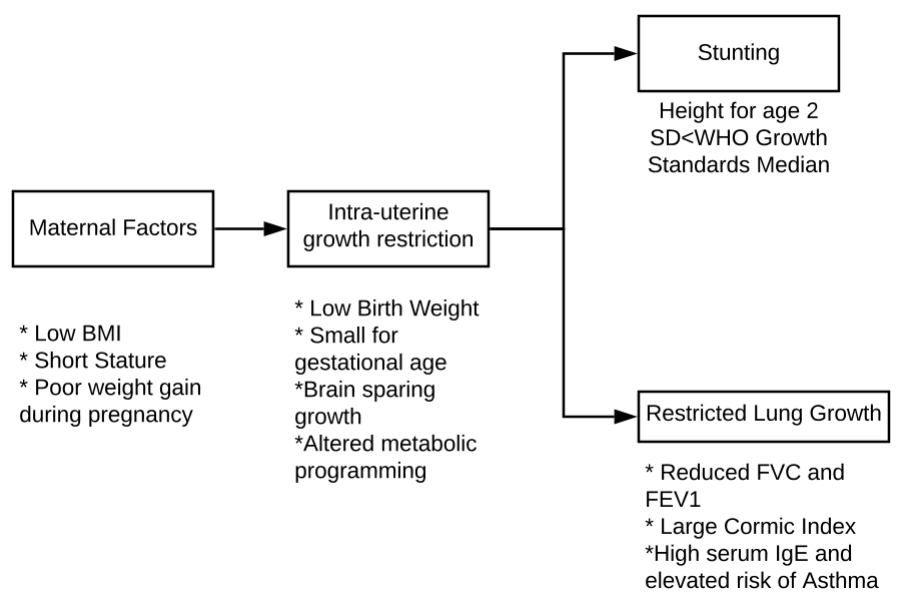
Intrauterine growth restriction contributes to both low lung function and stunting.

The foundation for compromised (‘brain sparing’) organ growth and metabolic dysfunction is laid during the perinatal period. According to the fetal origins hypothesis the fetus adapts itself in response to variations in nutrient and oxygen supply and its development is closely regulated by complex interactions between maternal nutritional status, endocrine and metabolic signals and placental development ^32^. ‘Size at birth’ and related derivatives such as small for gestational age (‘SGA’) reflect metabolic and anthropometric programming in the intrauterine environment (see Figure 1).

IUGR results in metabolic reprogramming during periods of rapid cell proliferation and differentiation. In later life, exposure to IUGR works synergistically with ante-natal factors such as malnutrition and infections during early life, to result in a compounded risk of stunting. Maternal anthropometric indicators of IUGR, such as short maternal stature, low body mass index and poor weight gain during pregnancy contribute to a higher risk of SGA and stunting in the child ^33– 38^.

It is well documented that that the timing of undernutrition determines the pattern of growth retardation. Babies with large heads are speculated to have grown more rapidly during early gestation such that their higher demand for nutrients during later gestation remains unmet ^39^. Early undernutrition results in small but normally proportioned animals, while later undernutrition results in selective organ damage. Babies who have experienced undernutrition in later gestation therefore have small lungs for their bodies ^40^.

Besides other consequences of disparate nutrient supply, thymus growth impairment during late gestation disrupts the differentiation of specific thymus-derived helper lymphocytes (Th) from Th2 to Th1, leading to exaggerated IgE responses and hyper responsive airways in later life ^41, 42^. This explains the association linking larger head circumference and increased serum IgE concentrations to the development of asthma in later life, while low birth weight is known to be associated with reduced FEV and FVC ^39, 43, 44^.

A study conducted by Todisco *et al.* compared the lung function of former pre-term and full-term children at 12.5 years of age and found higher rates of low lung function in the pre-term birth category compared to matched siblings delivered at term, indicating that lung function deficit at birth persists into early adolescence ^45^. This is because birth offsets one of the first and most profound gene environment interactions where the delivery of oxygen via the placenta is transferred to the lung, a process that is adversely affected by preterm birth. Evidence from expression profiling studies suggests that it is after the expression of developmental genes that genes involved in oxygen transport, genes coding for antioxidant species and genes involved in host defense are expressed, signaling a strong dependence on a developmentally mature and functional lung, which in preterm births is usually compromised. Additionally, supplemental oxygen therapy for preterm neonates not only causes inadvertent oxidative damage but also results in a highly simplified alveolar epithelium because of aberrant immune response. This aberrant response additionally suppresses angiogenic factors ^46^ interfering with healthy lung development. Maternal hypertension and pre-eclampsia, often implicated in pre term birth and low birth weight infants can be indirectly implicated in contributing to low lung function during childhood ^47, 48^.

In addition to direct effects such as a higher risk of preterm birth, compromised organ growth and stunting IUGR also exacerbates the adverse consequences of preterm delivery and postnatal hyperoxia ^49^. Preterm birth renders growth-restricted infants vulnerable to infections, leading to growth faltering, triggering a cycle of infection and undernutrition, hindering the attainment of maximum growth potential. IUGR directly results in brain sparing growth and restrictive lung growth but appears to set the foundation for stunting. The effects of IUGR and growth faltering in utero,when sustained through malnutrition and frequent infections leads to stunting as an outcome. This is an interesting point of convergence in the pathophysiology of both conditions.

### Malnutrition and anthropometry: Manifestations of stunting and low lung function

Nutritional insults throughout the life course initiate and sustain the pathophysiology of both stunting and restrictive lung growth. The Avon Longitudinal Study of Parents and Children reported positive associations between maternal intake of zinc and childhood FEV _1_ and FVC ^50^. FVC was found to be higher in children who were breastfed for 6 months or longer as compared to children breastfed between 2 to 4 months, among 4464 children embedded in a population-based prospective cohort ^51^. Postnatal vitamins A, E and D supplementation was observed to have the greatest effect on alveolar development and capillary growth, which are critical determinants of FVC ^52^. In growth restricted infants, as alveolar numbers continue to increase after birth, postnatal nutrition interventions may influence growth and affect the size of the adult lung ^53^.

Like restrictive lung function, growth faltering in stunting is compounded by suboptimal breastfeeding in the first months of life, a poor and unbalanced diet and/or insufficient vitamin and/or micronutrient intake and frequent infections during early childhood. In the Maternal and Child Undernutrition Group (a review of cohort studies from five low- and middle-income countries – including Brazil, Guatemala, India, Philippines and South Africa ^54^) SGA at birth and stunting were linked with short adult stature, reduced lean mass, which are also phenotypic correlates of low lung function.

### Manifestations in body composition and altered metabolic programming

**Figure 2.**
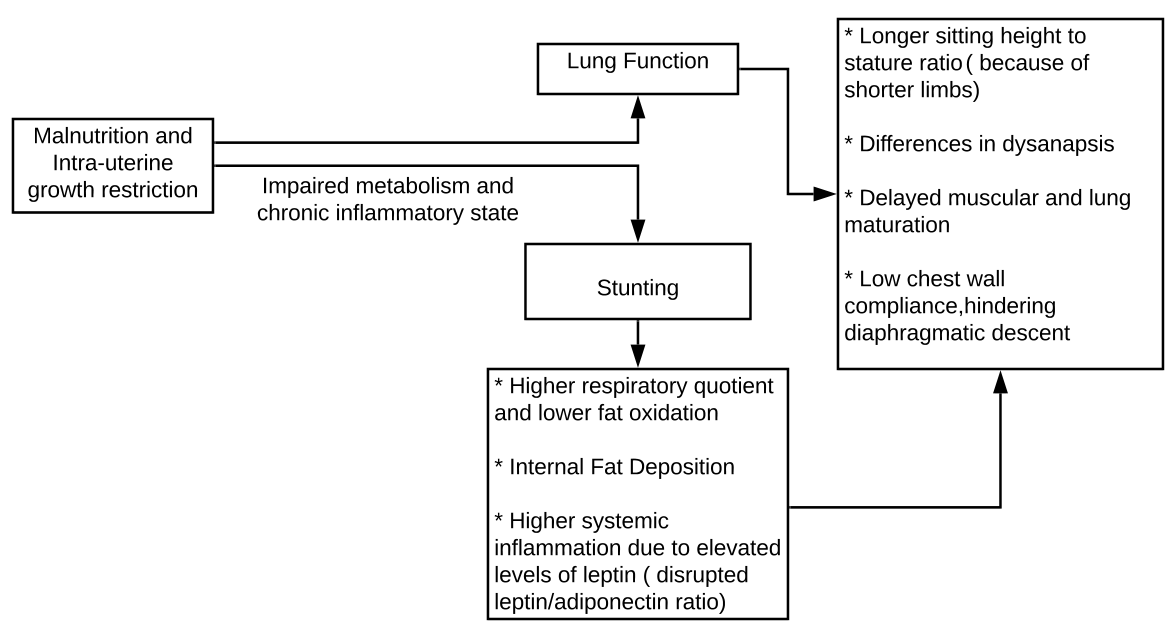
Stunting and restrictive lung function (FVC <80% of predicted) - anthropometric correlates.

Indeed, similar phenotypic adaptations conspicuous in anthropometry and body composition support the analogy between stunting and restricted lung growth (see Figure 2). For instance, stunted growth has disproportionate effects on FVC as compared to FEV _1_. This is because exposures implicated in stunting result in reduced lung growth and low FVC values, and due to their limited and indirect effect on FEV _1_, result in a high FEV _1_/FVC ratio. Therefore, stunting manifests with low values of FVC- indicating smaller lungs, as opposed to a smaller FEV1/FVC ratio, characteristic of airway obstruction ^21^. Analogous adaptations characterized by shorter limbs and sitting height are observed in both stunting and restrictive lung function ^55^. The positive association between age at peak adiposity and higher FVC, FEV _1_ and FEF _25-75_ implies that IUGR, followed by rapid weight gain during childhood results in poor lung function ^56^. Besides being shorter, stunted children have shorter leg length, resulting in a longer sitting-height-to stature ratio, which is known to influence population level differences in lung function ^57^.

With the exception of obese children exhibiting a reduction in static lung volume with degree of obesity, there exists a phase of transition from a positive to inverse association ^58– 63^. In the PIAMA birth cohort ^61^ (n=1288, at 12 years), high BMI and waist circumference were found to be associated with higher FVC, particularly in females. Girls with higher waist circumference and BMI at ages 8 and 12 had significantly higher FVC at age 12 than girls with normal BMI at both ages, suggesting that the inverse relationship between high BMI, waist circumference and FVC-FEV1 develops after age 12 ^64^.

The effect of stunted height on lung function growth is further compounded by maturational delays, particularly during the onset of adolescent growth spurt in stature. During puberty, dysynaptic growth appears to be more conspicuous in stunted children as compared to normal children, as stunted children are not only shorter but also more likely to exhibit delayed increment in muscular strength and lung maturation ^19, 65, 66^. However, while the phenotypic correlates of stunting recede due to rapid catch up growth during early childhood, indicators of restrictive lung function persist late into the life course.

### Air quality and environmental toxicants

**Figure 3.**
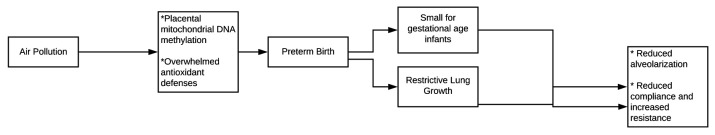
The role of air pollution in stunting and RLLF.

Inhalation of fine particles (particulate matter with diameter ≤2.5 μm; PM _2.5_) can induce oxidative stress and inflammation, and may contribute to onset of preterm labor and other adverse perinatal outcomes. This triggers a chain of events leading to SGA infants with poorly developed lungs (see Figure 3). Exposure to environmental toxicants is another factor common to the origin of both low lung function and stunting. It impacts health through both inhalation and trans-placental transmission *in utero* ^67^. Low birth weight is also an independent risk factor for stunting, particularly in developing nations with both high air pollution and malnutrition contributing to IUGR ^68^.

Low birth weight was higher among women who delivered in facilities where PM _2.5_ concentrations were above the median (i.e., >12.0 μg/m ^3^) compared with women delivering at facilities with average PM _2.5_ levels <6.3 μg/m ^3^. In China, the country with the largest range of PM _2.5_ exposure levels, both preterm birth and LBW were significantly higher among women with estimated exposure to at least 36.5 μg/m ^3^ of PM _2.5_ compared with women in the lowest quartile of exposure (<12.5 μg/m ^3^) ^69^. The ENVIRONAGE birth cohort too reported an association between *in utero* PM 2.5 exposure and placental mitochondrial DNA methylation in 381 mother-newborn pairs ^70^.

In addition to mechanisms operating through pre-term birth, exposure to air pollution also directly affects lung function growth. In a Californian study in 232 asthmatic children, fetal exposure to PM _10_ during the first trimester of pregnancy was found to be associated with a lower peak expiratory flow volume between ages 6–11 years ^71^. A 90 mL lower FEV _1_ at 5 years was observed in the Krakow birth cohort, comprising 176 exposed children of non-smoking mothers ^72^, while a 60mL reduction in FEV _1_ was found in Swedish children exposed to higher concentrations of PM _10_ during the first year of life. These children were likely to have FEV _1_ and FVC less than the lower limit of normal at age 16. Children of the same cohort exhibited higher peripheral airway resistance from impulse oscillometry (R _5_–R _20_) at age 16 ^73^.

In non-cigarette smoking women with lifelong biomass exposure, a direct link between childhood exposure to PM and an increased susceptibility to adult respiratory disease (including COPD) was observed ^74^. In experimental studies conducted on mice with either pre or postnatal exposure to traffic-related PM, significant alteration of alveolar structure and changes in the elastic properties of the lung were observed ^75^.

Newborns exposed to PM _10_ *in utero* exhibited a higher oxygen demand, indicated by higher minute ventilations and tidal flows. These changes were similar to those in premature infants with broncho-pulmonary dysplasia, infants with smoking mothers and in animal models of pre-natal nicotine exposure and were also indicative of increased airway resistance (smaller airways), decreased compliance (smaller/stiffer airways) and disruption of factors that directly influence control of breathing. Air pollution induced oxidative stress and localized or systemic inflammation in the mother could affect permeability of the blood-air barrier, leading to an increase in fetal breathing movements and reduced alveolarisation. Reduced alveolarisation could also be a result of systemic inflammation, which disrupts placental blood flow and affects the nutrient transfer to the fetus, influencing intrauterine growth and future lung function ^76, 77^.

Post birth, pre-term or small for gestational age children in developing nations traditionally experiencing the effects of intergenerational malnutrition, are also more likely to be exposed to a higher level of ambient PM 2.5 both *in utero* and during early childhood, and exhibit higher risk for anthropometric failure, even after accounting for various confounding characteristics.

### Maternal smoking and tobacco consumption

*In utero* exposure to nicotine remains the single, most important and potentially preventable insult to the developing lung. It is a major cause of sudden infant death, LBW, preterm delivery and IUGR ^78^. In 2015, out of 933 million daily smokers, 5.4% were women, while 72.5% of pregnant women who smoke, were daily smokers throughout their pregnancies and around 2% of women smoking throughout their pregnancies resided in South East Asia and Africa ^79, 80^.

In addition to IUGR and low birth weight, maternal smoking was found to increase the risk of COPD in offspring by 1.7, and in terms of airflow limitation was equivalent to 10 years of personal smoking by the offspring ^81^. The effect of smoking on lung function may transcend generations, as Grand-maternal smoking not only increases the risk of maternal asthma, but also raises the risk of asthma in her offspring even if the mother herself does not smoke.

Gender is an effect modifier in the association between *in utero*/postnatal exposure to secondhand smoke, with a stronger association in males than in females. *In utero*/postnatal exposure to second hand smoke results in a 64.6% odds of reduced FVC in males and a 21.6% odds of reduced FVC in females ^82^.

The immediate effects of tobacco exposure are difficult detect because children exposed to tobacco smoke do not necessarily manifest reduced lung function or increased propensity for respiratory morbidity possibly owing to differences in maternal and fetal susceptibility ^83^. It is also difficult to distinguish between the effects of pre and postnatal tobacco exposure because women who smoke during pregnancy continue to do so after childbirth.

**Figure 4.**
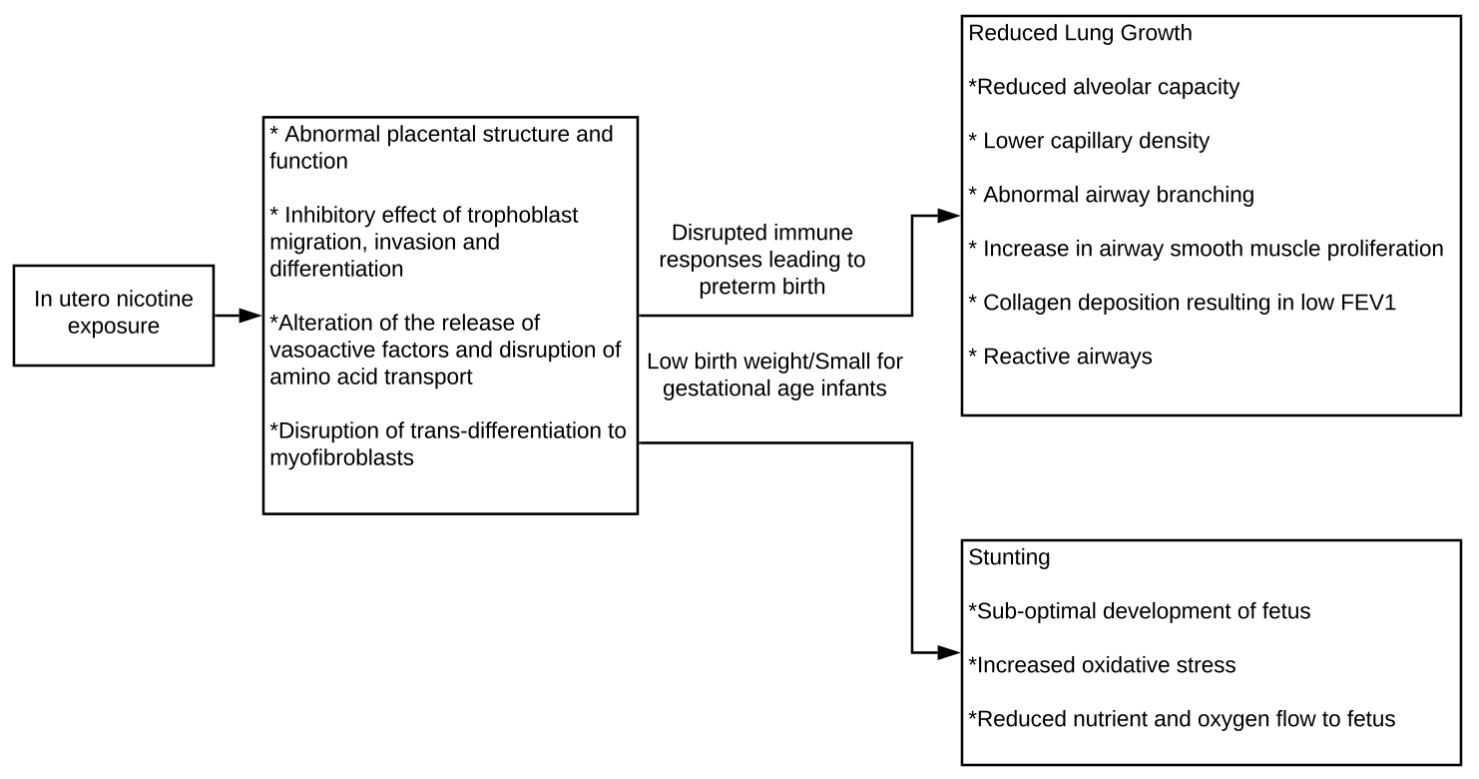
*In utero* nicotine exposure, RLLF and Stunting ^78, 81– 83, 98^.

However, it is clear that multiple inflammatory insults from tobacco exposure reduce airway caliber and disrupt fetal immune responses inducing prematurity and low birth weight, resulting in growth restricted infants ^84^. As IUGR and size at birth predict risk of stunting and restrictive lung function, controlling maternal smoking may influence both outcomes substantially (see Figure 4).

### The impoverished gut

Stunted children inhabit unhygienic settings and live in conditions of acute deprivation where environmental enteric dysfunction (EED) is prevalent ^85^. EED is a result of sustained and frequent low inoculum exposure to a wide range of pathogens, mostly through contaminated food and water. The resulting low grade infection causes both systemic and gut inflammation leading to intestinal leakiness ,heightened permeability, nutrient malabsorption and disrupted immunomodulation ^86, 87^. Frequent infections, vaccine failures and chemotherapy lead to a disruption in the homeostasis of the gut microbiota, a phenomenon referred to as dysbiosis ^88^.

The gut microbial community possesses enzymatic machinery for assimilating a variety of dietary nutrients leading to the release of multi-functional metabolites in the host. EED compromises gut integrity and when coupled with immaturity and dysbiosis of the gut microbiome, hampers nutrient assimilation ^89– 91^. This leads to a pattern of growth faltering and recurrent infections leading to a decline in length-for-age Z scores, particularly among children between 18–24 months of age.

Although the mature gastrointestinal tract and the respiratory tract (RT) have different environments and functions, they have the same embryonic origin and therefore share structural similarities. Thus, similar mechanisms operating bi-directionally along the gut-lung axis allow GI microbiota to play a key role in immune adaptation and initiation at other distal mucosal sites such as the lung ^92^.

This cross-talk along the gut lung axis happens during early development, possibly during the first two years of life, which are critical to the stabilization of an individual’s microbiome. This hypothesis is supported by the existence of a strong correlation between low microbial diversity in the gut during early infancy and an asthmatic phenotype in childhood and the simultaneous manifestation of both respiratory and GI disease symptoms during adulthood ^92– 94^. An asthmatic phenotype during childhood was seen to be associated with an elevated risk of COPD ^95^ and lower values of FEV1,FVC during adulthood (when compared to control groups without asthma during childhood) ^96^. These associations offer subtle insights into the role of gut microbiota in influencing long term adult lung function, via other indirect influences. 

**Figure 5.**
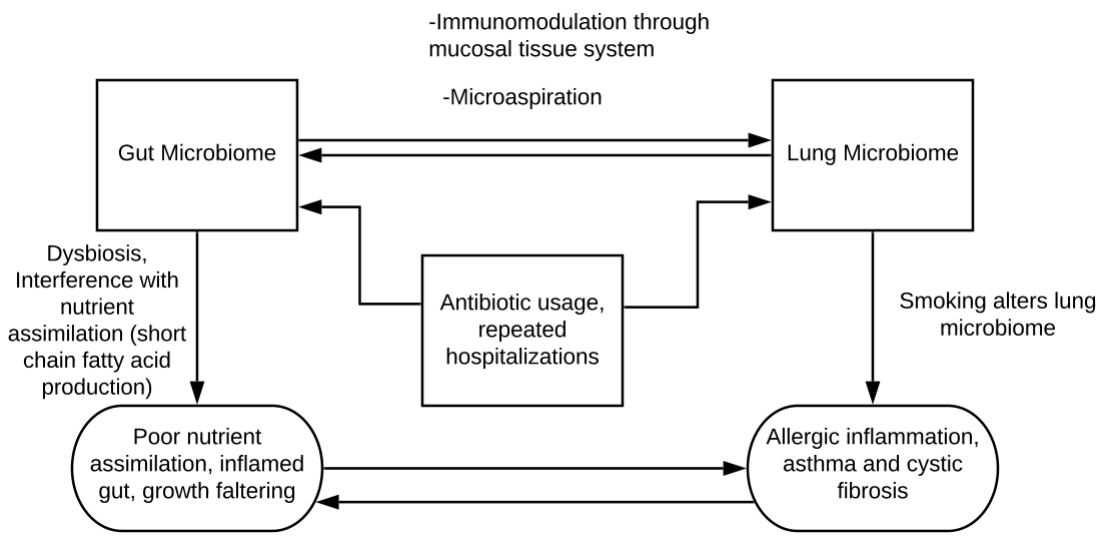
How the impoverished gut may mediate stunting and low lung function.

EED further amplifies the effects of growth faltering and poor lung development in the developing world by reducing the efficacy of oral vaccines, possibly even leading to vaccine failure (see Figure 5). Among Bangladeshi infants, EED was linked to the reduced efficacy of oral polio and rotavirus vaccines ^97^. Barriers to nutrient absorption and disrupted immunomodulation thus affect both growth and lung development. Although the gut–lung axis is only beginning to be understood, emerging evidence indicates that there is potential for manipulation of the gut microbiota in the treatment of lung diseases.

The effect of gut microbiome on nutrient assimilation is relevant to the implementation of oral vaccination and nutrition programs in the developing world where EED is rampant.

### Genetic and molecular modulators of perinatal lung maturation

The genetic and epigenetic correlates of both conditions largely appear to encode physiological responses to early nutritional and environmental insults.

Around 50 genes for lung function and 13 genes influencing the indicators of stunting were identified, and seen to influence similar early developmental pathways and nutrient absorption Of these, three genes— *FGF21* (cellular proliferation, survival, migration, and differentiation), *FUT2* (cell-cell interaction, interaction with intestinal microbiota) and *IGF1*/ *IGF2* (growth promotion, 2DG transport and glycogen synthesis in osteoblasts)—were found to be common to both conditions (see Table 1). The individual effect sizes of these genetic and epigenetic modulators is small (known SNPs for FVC account for only 14.3% of variation in heritability).

**Table 1.** Genes implicated in stunting and low lung function. (Lung function ^100– 105^, stunting ^106– 109^).

Gene (Lung Function) ( FEV/FVC, FVC)	P Value	Function	Gene (Stunting)	P Value	Function
**IGF-1**	2.19E-13	Development and differentiation of many types of lung cells, including airway basal cells, club cells, alveolar epithelial cells, and fibroblasts.	**IGF1/IGF-2**	5.22E-22	IGFs are involved in the proliferation, differentiation and apoptosis of fetal cells in vitro and the IGF serum concentration is closely correlated with fetal growth and length.
**FGF21**	-	Inhibits pulmonary fibrosis through activating Nrf-2 pathway, subsequently suppressing oxidative stress, inhibiting ECM deposition and pulmonary fibrogenesis	**FGF21**	7.9E−09	Decreased protein intake, increased carbohydrate intake, and decreased fat intake after adjusting for body mass index
**FUT2**	0.00	Generating the H-type antigen in saliva and on digestive and respiratory epithelia	**FUT2**	8.00E-14	Involved in histo-blood group antigen production, Diarrhea by one year of age
**BMP6**	1.45E-06	Encoded ligand for transforming growth factor,ERK signalling	**NTN5/SEC1P**	7.00E-11	Diarrhea by one year of age
**EFEMP1**	1.45E-06	Calcium ion binding and epidermal growth factor receptor binding,cell adhesion and migration. May function as a negative regulator of chondrocyte differentiation	**CREM84,85**	6.05E-09	Susceptibility to susceptibility to E. histolytica disease
**HSD17B12**	3.70E-05	Long-chain fatty acids elongation cycle	**H6PD**	3.19E-07	carbohydrate binding and glucose-6- phosphate dehydrogenase activity
**PRDM11**	4.80E-05	Nucleic acid binding and methyltransferase activity	**RSRC1-SHOX2**	2.14E-09	Transcriptional regulation, constitutive and alternative pre-mRNA splicing
**WWOX**	2.04E-05	Regulation of a wide variety of cellular functions, such as protein degradation, transcription and RNA splicing	**PPP2R2A**	4.29E-05	Negative control of cell growth and division,protein serine/threonine phosphatase activity
**KCNJ2**	3.73E-05	Probably participates in establishing action potential waveform and excitability of neuronal and muscle tissues,	**FTO**	1.42E-08	Total percentage of carbohydrate intake,childhood obesity ,
**LCT**	2.18E-08	Hydrolase activity, hydrolyzing O-glycosyl compounds and lactase activity	**TANK**	1.00E-05	Decreased fat intake, increased carbohydrate intake, and marginally decreased protein intake
**FGF10**	1.49E-06	Embryonic development, cell growth, morphogenesis, tissue repair	**FADS1, FAD2, FADS3**	4.20E-09	Breast milk fatty acid composition,regulating immunity and inflammation
**TMEM163**	3.60E-07	May bind zinc and other divalent cations and recruit them to vesicular organelles	**RBP4**	-	Retinol Binding Protein 4, specific transport protein for vitamin A in the circulation
**FAM13A**	2.90E-03	putative role in signal transduction, Signaling by GPCR and Signaling by Rho GTPases	**TMEM18**	2.03E-04	vesicle transport in exocrine cells and Sertoli cells

The genes common to both forced vital capacity and stunting were found to be largely associated with early development (IGF1,BMP6), morphogenesis(IGF1) and nutritional insults due to recurrent GI infections (BMP6,FUT2) . (See Table 1) 

While the small heritability of associated genes did not lend much support to the analogy, identification of unique SNPs with high heritability may be useful in paving the way for community profiling and the mapping of appropriate interventions to communities.

## Conclusion

**Figure 6.**
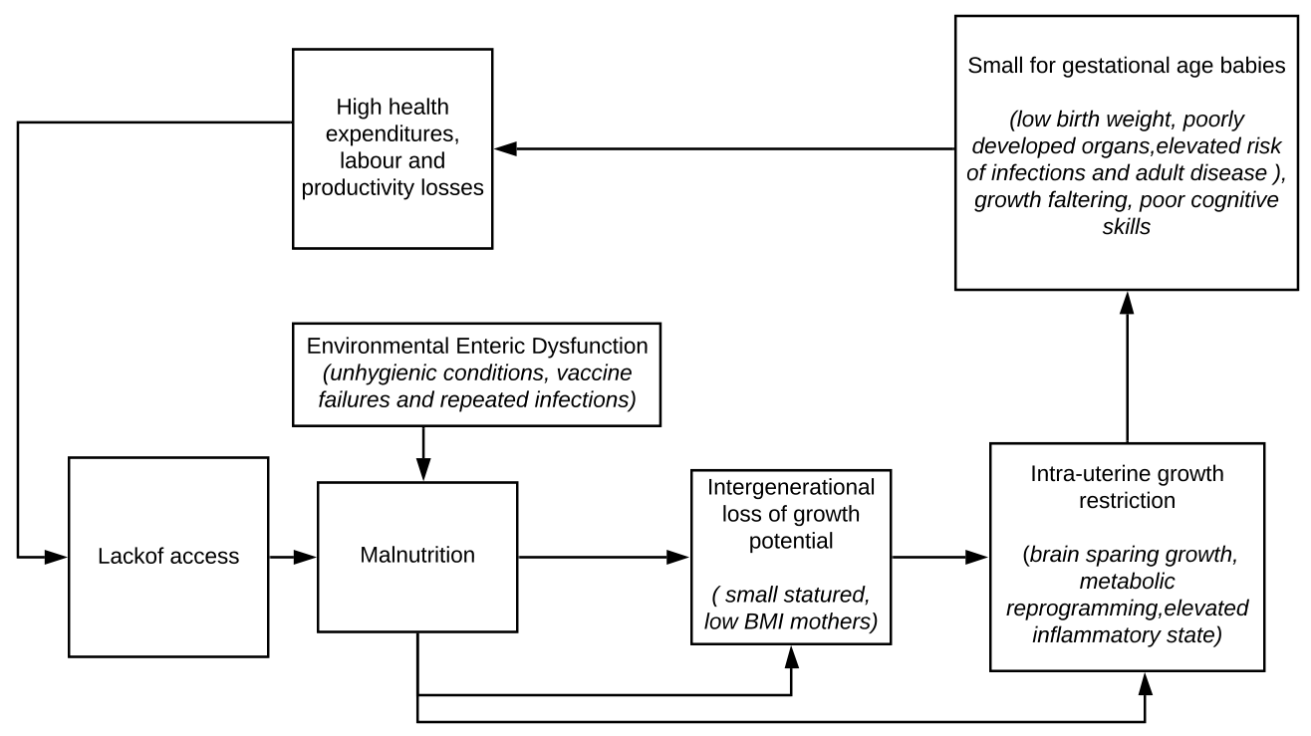
Intergenerational effects of malnutrition and inequality.

While IUGR is central to the pathophysiology of both stunting and compromised lung growth, malnutrition, mediated by several complex factors, appears to be the true point of convergence (see Figure 6). Although malnutrition may manifest in several ways, WHO maintains that the path to prevention remains identical across populations. Major preventive measures may include: adequate maternal nutrition ranging from the perinatal period to lactation, optimal breast feeding during the first two years of life, healthy childhood nutrition, sanitation and safe physical activity ^99^. In addition to multi sectoral collaborations, design of appropriate interventions, embedding NCD impact evaluation into maternal and child health programs is crucial to addressing rapid epidemiological transitions in the developing world.

Our inability to maintain stringent inclusion criteria of human randomized controlled trials in areas of nutrition, vaccination, tobacco cessation and environmental health across populations in developing nations is representative of the absence of necessary research to guide interventions in this area. This necessitated a narrative review design to present an updated perspective and not to directly guide clinical practice.

The correlation between Socio Demographic Index and indicators of both chronic and infectious diseases reflects the need to understand heterogeneity in lung function and linear growth patterns in the context of socioeconomic variations that determine nutritional and environmental exposures, access to sanitary living conditions and inter-generational patterns of growth faltering. Identifying highly heritable genetic variants, which could potentially mediate response to interventions, might serve as genetic signatures unique to communities. These inputs could assist in tailoring interventions for communities by capturing meaningful environmental influences in addition to ethnic differences.

Creation of proxy scores for communities incorporating epidemiological transition levels, heritability of traits associated with disease, responses to existing programs and, metabolic health and growth trajectories could aid in mapping communities to appropriate health interventions. Further research is needed in utilizing existing data sources, assigning weights to individual components and generating comprehensive scores useful for community profiling.

## Data availability

### Underlying data

All data underlying the results are available as part of the article and no additional source data are required.

### Extended data

Figshare: Review Data (GBD).csv. https://doi.org/10.6084/m9.figshare.12278129 ^110^.

This file contains the collated data used examine the relationship between SDI, risk factors and diseases (in the section “ *Higher burden of stunting and low lung function in the developing world indicates the role of socioeconomic influences*”). Data were originally obtained from the GBD visualization tool, originally available at https://vizhub.healthdata.org/gbd-compare/ ^15^.

Extended data are available under the terms of the Creative Commons Attribution 4.0 International license (CC-BY 4.0).

## References

[1] Kannel W Hubert H Lew E: Vital capacity as a predictor of cardiovascular disease: The Framingham study. *Am Heart J.* 1983;105(2):311–315. 10.1016/0002-8703(83)90532-x 6823813

[2] Mannino DM: Respiratory disease in 2010: looking to the past will prepare us for the future. *Thorax.* 2010;65(6):469–471. 10.1136/thx.2010.137570 20522839

[3] Huang S Vasquez MM Halonen M: Asthma, airflow limitation and mortality risk in the general population. *Eur Respir J.* 2015;45(2):338–346. 10.1183/09031936.00108514 25323227 PMC4683019

[4] Cuttica MJ Colangelo LA Dransfield MT: Lung Function in Young Adults and Risk of Cardiovascular Events Over 29 Years: The CARDIA Study. *J Am Heart Assoc.* 2018;7(24):e010672. 10.1161/JAHA.118.010672 30561252 PMC6405620

[5] Agrawal A: Developing “Vital Capacity” in Cardiovascular Risk Assessment. *Circulation.* 2019;140(16):1291–1292. 10.1161/CIRCULATIONAHA.119.041745 31609660

[6] Guerra S Sherrill DL Venker C: Morbidity and mortality associated with the restrictive spirometric pattern: a longitudinal study. *Thorax.* 2010;65(6):499–504. 10.1136/thx.2009.126052 20522846 PMC3036842

[7] Agrawal A Aggarwal M Sonnappa S: Ethnicity and spirometric indices: hostage to tunnel vision? *Lancet Respir Med.* 2019;7(9):743–744. 10.1016/S2213-2600(19)30204-8 31208952 PMC6906084

[8] World Health Organization, U N C: WHO child growth standards and the identification of severe acute malnutrition in infants and children. 2009 Reference Source 24809116

[9] Duong M Islam S Rangarajan S: Global differences in lung function by region (PURE): an international, community-based prospective study. *Lancet Respir Med.* 2013;1(8):599–609. 10.1016/S2213-2600(13)70164-4 24461663

[10] Obaseki DO Erhabor GE Awopeju OF: Reduced Forced Vital Capacity in an African Population. Prevalence and Risk Factors. *Ann Am Thorac Soc.* 2017;14(5):714–721. 10.1513/AnnalsATS.201608-598OC 28244800 PMC5427737

[11] Mannino DM \ McBurnie MA Tan W: Restricted spirometry in the Burden of Lung Disease Study. *Int J Tuberc Lung Dis.* 2012;16(10):1405–11. 10.5588/ijtld.12.0054 22863565

[12] Martinez CH Mannino DM Curtis JL: Socioeconomic Characteristics Are Major Contributors to Ethnic Differences in Health Status in Obstructive Lung Disease: An Analysis of the National Health and Nutrition Examination Survey 2007-2010. *Chest.* 2015;148(1):151–158. 10.1378/chest.14-1814 25633478 PMC4493871

[13] Burney P Jithoo A Kato B: Chronic obstructive pulmonary disease mortality and prevalence: the associations with smoking and poverty--a BOLD analysis. *Thorax.* 2014;69(5):465–73. 10.1136/thoraxjnl-2013-204460 24353008 PMC3995258

[14] Singhal A: Long-Term Adverse Effects of Early Growth Acceleration or Catch-Up Growth. *Ann Nutr Metab.* 2017;70(3):236–240. 10.1159/000464302 28301849

[15] IHME: Global Burden of Disease Study 2017 (GBD 2017). 2019 Reference Source

[16] Bui DS Burgess JA Lowe AJ: Childhood Lung Function Predicts Adult Chronic Obstructive Pulmonary Disease and Asthma-Chronic Obstructive Pulmonary Disease Overlap Syndrome. *Am J Respir Crit Care Med.* 2017;196(1):39–46. 10.1164/rccm.201606-1272OC 28146643

[17] DeBoer MD Lima AAM Oría RB: Early childhood growth failure and the developmental origins of adult disease: do enteric infections and malnutrition increase risk for the metabolic syndrome? *Nutr Rev.* 2012;70(11):642–53. 10.1111/j.1753-4887.2012.00543.x 23110643 PMC3493112

[18] Mbwana HA Kinabo J Lambert C: Factors influencing stunting among children in rural Tanzania: an agro-climatic zone perspective. *Food Secur.* 2017;9:1157–1171. 10.1007/s12571-017-0672-4

[19] Weitz CA Garruto RM: Stunting and the Prediction of Lung Volumes Among Tibetan Children and Adolescents at High Altitude. *High Alt Med Biol.* 2015;16(4):306–317. 10.1089/ham.2015.0036 26397381 PMC4685485

[20] Tarazona-Meza CE Nicholson A Romero KM: Household food insecurity is associated with asthma control in Peruvian children living in a resource-poor setting. *J Asthma.* 2019;1–8. 10.1080/02770903.2019.1648506 31418600 PMC7079231

[21] Quanjer PH Stanojevic S Stocks J: Changes in the FEV _1_/FVC ratio during childhood and adolescence: an intercontinental study. *Eur Respir J.* 2010;36(6): 1391–1399. 10.1183/09031936.00164109 20351026

[22] Whittaker AL Sutton AJ Beardsmore CS: Are ethnic differences in lung function explained by chest size? *Arch Dis Child Fetal Neonatal Ed.* 2005;90(5):F423–F428. 10.1136/adc.2004.062497 15871993 PMC1721951

[23] Saad NJ Patel J Minelli C: Explaining ethnic disparities in lung function among young adults: A pilot investigation. *PLoS One.* 2017;12(6):e0178962. 10.1371/journal.pone.0178962 28575113 PMC5456386

[24] Jacobs DR Jr Nelson ET Dontas AS: Are race and sex differences in lung function explained by frame size? The CARDIA Study. *Am Rev Respir Dis.* 1992;146(3):644–649. 10.1164/ajrccm/146.3.644 1519841

[25] Olden K White SL: Health-related disparities: influence of environmental factors. *Med Clin North Am.* 2005;89(4):721–738. 10.1016/j.mcna.2005.02.001 15925646

[26] Quanjer PH: Lung function, genetics and socioeconomic conditions. *Eur Respir J.* 2015;45(6):1529–1533. 10.1183/09031936.00053115 26028617

[27] Prendergast AJ Humphrey JH: The stunting syndrome in developing countries. *Paediatr Int Child Health.* 2014;34(4):250–265. 10.1179/2046905514Y.0000000158 25310000 PMC4232245

[28] Sonnappa S Lum S Kirkby J: Disparities in pulmonary function in healthy children across the Indian urban-rural continuum. *Am J Respir Crit Care Med.* 2015;191(1): 79–86. 10.1164/rccm.201406-1049OC 25412016 PMC4299630

[29] Sorkness R: Nature, Nurture, and Lung Volumes. *Am J Respir Crit Care Med.* 2015;191(1): 11–12. 10.1164/rccm.201412-2156ED 25551344

[30] Sonnappa S Lum S Kirkby J: Disparities in pulmonary function in healthy children across the Indian urban-rural continuum. *Am J Respir Crit Care Med.* 2015;191(1):79–86. 10.1164/rccm.201406-1049OC 25412016 PMC4299630

[31] Bommer C Vollmer S Subramanian SV: How socioeconomic status moderates the stunting-age relationship in low-income and middle-income countries. *BMJ Glob Health.* 2019;4(1):e001175. 10.1136/bmjgh-2018-001175 30899561 PMC6407538

[32] Eriksson JG: Developmental Origins of Health and Disease – from a small body size at birth to epigenetics. *Ann Med.* 2016;48(6):456–467. 10.1080/07853890.2016.1193786 27268105

[33] de Onis M Blössner M Villar J: Levels and patterns of intrauterine growth retardation in developing countries. *Eur J Clin Nutr.* 1998;52 Suppl 1:S5–15. 9511014

[34] Neufeld LM Haas JD Grajéda R: Changes in maternal weight from the first to second trimester of pregnancy are associated with fetal growth and infant length at birth. *Am J Clin Nutr.* 2004;79(4): 646–652. 10.1093/ajcn/79.4.646 15051610

[35] Addo OY Stein AD Fall CH: Maternal Height and Child Growth Patterns. *J Pediatr.* 2013;163(2):549–554.e1. 10.1016/j.jpeds.2013.02.002 23477997 PMC3711792

[36] Monden CWS Smits J: Maternal height and child mortality in 42 developing countries. *Am J Hum Biol.* 2009;21(3):305–311. 10.1002/ajhb.20860 19107903

[37] Ozaltin E Hill K Subramanian SV: Association of Maternal Stature With Offspring Mortality, Underweight, and Stunting in Low- to Middle-Income Countries. *JAMA.* 2010;303(15):1507–16. 10.1001/jama.2010.450 20407060 PMC3100588

[38] Subramanian SV Ackerson LK Smith GD: Association of Maternal Height With Child Mortality, Anthropometric Failure, and Anemia in India. *JAMA.* 2009;301(16):1691–701. 10.1001/jama.2009.548 19383960 PMC3095774

[39] Lopuhaä CE Roseboom TJ Osmond C: Atopy, lung function, and obstructive airways disease after prenatal exposure to famine. *Thorax.* 2000;55(7):555–561. 10.1136/thorax.55.7.555 10856314 PMC1745806

[40] Shaheen SO Barker DJ Holgate ST: Do lower respiratory tract infections in early childhood cause chronic obstructive pulmonary disease? *Am J Respir Crit Care Med.* 1995;151(5):1649–51; discussion 1651-2. 10.1164/ajrccm/151.5_Pt_1.1649 7735628

[41] Matson AP Zhu L Lingenheld EG: Maternal Transmission of Resistance to Development of Allergic Airway Disease. *J Immunol.* 2007;179(2):1282–1291. 10.4049/jimmunol.179.2.1282 17617621 PMC3155847

[42] Murdoch JR Lloyd CM: Chronic inflammation and asthma. *Mutat Res.* 2010;690(1–2):24–39. 10.1016/j.mrfmmm.2009.09.005 19769993 PMC2923754

[43] Dezateux C Stocks J: Lung development and early origins of childhood respiratory illness. *Br Med Bull.* 1997;53(1):40–57. 10.1093/oxfordjournals.bmb.a011605 9158283

[44] Dezateux C Lum S Hoo AF: Low birth weight for gestation and airway function in infancy: exploring the fetal origins hypothesis. *Thorax.* 2004;59(1):60–6. 14694251 PMC1758850

[45] Todisco T de Benedictis FM Iannacci L: Mild prematurity and respiratory functions. *Eur J Pediatr.* 1993;152(1):55–58. 10.1007/BF02072517 8444206

[46] Thébaud B Abman SH: Bronchopulmonary Dysplasia: Where Have All the Vessels Gone? Roles of Angiogenic Growth Factors in Chronic Lung Disease. *Am J Respir Crit Care Med.* 2007;175(10):978–985. 10.1164/rccm.200611-1660PP 17272782 PMC2176086

[47] Wilmink FA den Dekker HT de Jongste JC: Maternal blood pressure and hypertensive disorders during pregnancy and childhood respiratory morbidity: the Generation R Study. *Eur Respir J.* 2018;52(5):1800378. 10.1183/13993003.00378-2018 30309974

[48] Gutiérrez-Delgado RI Barraza-Villarreal A Escamilla-Núñez C: Effect of omega-3 fatty acids supplementation during pregnancy on lung function in preschoolers: a clinical trial. *J Asthma.* 2019;56(3):296–302. 10.1080/02770903.2018.1452934 29617210

[49] Malhotra A Allison BJ Castillo-Melendez M: Neonatal Morbidities of Fetal Growth Restriction: Pathophysiology and Impact. *Front Endocrinol (Lausanne).* 2019;10:55. 10.3389/fendo.2019.00055 30792696 PMC6374308

[50] Bédard A Northstone K Henderson AJ: Maternal intake of sugar during pregnancy and childhood respiratory and atopic outcomes. *Eur Respir J.* 2017;50(1):1700073. 10.1183/13993003.00073-2017 28679610 PMC5540678

[51] van Meel ER den Dekker HT Elbert NJ: A population-based prospective cohort study examining the influence of early-life respiratory tract infections on school-age lung function and asthma. *Thorax.* 2018;73(2):167–173. 10.1136/thoraxjnl-2017-210149 29101282 PMC6485606

[52] Checkley W West KP Jr Wise RA: Maternal Vitamin A Supplementation and Lung Function in Offspring. *N Engl J Med.* 2010;362(19):1784–1794. 10.1056/NEJMoa0907441 20463338

[53] Packard CJ Bezlyak V McLean JS: Early life socioeconomic adversity is associated in adult life with chronic inflammation, carotid atherosclerosis, poorer lung function and decreased cognitive performance: a cross-sectional, population-based study. *BMC Public Health.* 2011;11: 42. 10.1186/1471-2458-11-42 21241479 PMC3032683

[54] Black RE Victora CG Walker SP: Maternal and child undernutrition and overweight in low-income and middle-income countries. *Lancet.* 2013;382(9890):427–451. 10.1016/S0140-6736(13)60937-X 23746772

[55] Metcalfe NB Monaghan P: Compensation for a bad start: grow now, pay later? *Trends Ecol Evol.* 2001;16(5):254–260. 10.1016/s0169-5347(01)02124-3 11301155

[56] Byberg KK Mikalsen IB Eide GE: The associations between weight-related anthropometrics during childhood and lung function in late childhood: a retrospective cohort study. *BMC Pulm Med.* 2018;18(1): 10. 10.1186/s12890-017-0567-3 29351745 PMC5775530

[57] Quanjer PH Kubota M Kobayashi H: Secular Changes in Relative Leg Length Confound Height-Based Spirometric Reference Values. *Chest.* 2015;147(3):792–797. 10.1378/chest.14-1365 25254426

[58] Curry BA Blizzard CL Schmidt MD: Longitudinal Associations of Adiposity With Adult Lung Function in the Childhood Determinants of Adult Health (CDAH) Study. *Obesity (Silver Spring).* 2011;19(10):2069–2075. 10.1038/oby.2011.47 21436794

[59] Ochs-Balcom HM Grant BJB Muti P: Pulmonary Function and Abdominal Adiposity in the General Population. *Chest.* 2006;129(4):853–862. 10.1378/chest.129.4.853 16608930

[60] Cibella F Bruno A Cuttitta G: An Elevated Body Mass Index Increases Lung Volume but Reduces Airflow in Italian Schoolchildren. *PLoS One.* 2015;10(5):e0127154. 10.1371/journal.pone.0127154 25970463 PMC4430514

[61] Khan S Little J Chen Y: Relationship Between Adiposity and Pulmonary Function in School-Aged Canadian Children. *Pediatr Allergy Immunol Pulmonol.* 2014;27(3):126–132. 10.1089/ped.2014.0349 25276486 PMC4170807

[62] Chen Y Rennie D Cormier Y: Waist circumference associated with pulmonary function in children. *Pediatr Pulmonol.* 2009;44(3):216–221. 10.1002/ppul.20854 19205050

[63] Suresh S O’Callaghan M Sly PD: Impact of Childhood Anthropometry Trends on Adult Lung Function. *Chest.* 2015;147(4):1118–1126. 10.1378/chest.14-0698 25340561

[64] Bekkers MB Wijga AH Gehring U: BMI waist circumference at 8 and 12 years of age and FVC and FEV1 at 12 years of age; the PIAMA birth cohort study. *BMC Pulm Med.* 2015;15:39. 10.1186/s12890-015-0032-0 25896340 PMC4409985

[65] Smith JR Emerson SR Kurti SP: Lung volume and expiratory flow rates from pre- to post-puberty. *Eur J Appl Physiol.* 2015;115(8):1645–1652. 10.1007/s00421-015-3149-1 25761732

[66] Zeman KL Bennett WD: Growth of the small airways and alveoli from childhood to the adult lung measured by aerosol-derived airway morphometry. *J Appl Physiol.* 2006;100(3):965–971. 10.1152/japplphysiol.00409.2005 16357074

[67] Goyal N Canning D: Exposure to Ambient Fine Particulate Air Pollution in Utero as a Risk Factor for Child Stunting in Bangladesh. *Int J Environ Res Public Health.* 2017;15(1):22. 10.3390/ijerph15010022 29295507 PMC5800122

[68] Aryastami NK Shankar A Kusumawardani A: Low birth weight was the most dominant predictor associated with stunting among children aged 12–23 months in Indonesia. *BMC Nutr.* 2017;3:16 10.1186/s40795-017-0130-x

[69] Fleischer NL Merialdi M van Donkelaar A: Outdoor Air Pollution, Preterm Birth, and Low Birth Weight: Analysis of the World Health Organization Global Survey on Maternal and Perinatal Health. *Environ Health Perspect.* 2014;122(4):425–430. 10.1289/ehp.1306837 24508912 PMC3984219

[70] Janssen BG Byun HM Gyselaers W: Placental mitochondrial methylation and exposure to airborne particulate matter in the early life environment: An ENVIR ON AGE birth cohort study. *Epigenetics.* 2015;10(6):536–544. 10.1080/15592294.2015.1048412 25996590 PMC4623402

[71] Mortimer K Neugebauer R Lurmann F: Air Pollution and Pulmonary Function in Asthmatic Children. *Epidemiology.* 2008;19(4):550–557. 10.1097/EDE.0b013e31816a9dcb 18520616

[72] Jedrychowski WA Perera FP Maugeri U: Effect of prenatal exposure to fine particulate matter on ventilatory lung function of preschool children of non-smoking mothers. *Paediatr Perinat Epidemiol.* 2010;24(5):492–501. 10.1111/j.1365-3016.2010.01136.x 20670230 PMC3761386

[73] Schultz ES Gruzieva O Bellander T: Traffic-related Air Pollution and Lung Function in Children at 8 Years of Age. *Am J Respir Crit Care Med.* 2012;186(12):1286–1291. 10.1164/rccm.201206-1045OC 23103735

[74] Grigg J: Particulate Matter Exposure in Children: Relevance to Chronic Obstructive Pulmonary Disease. *Proc Am Thorac Soc.* 2009;6(7):564–569. 10.1513/pats.200905-026RM 19934350

[75] Mauad T Rivero DHR de Oliveira RC: Chronic Exposure to Ambient Levels of Urban Particles Affects Mouse Lung Development. *Am J Respir Crit Care Med.* 2008;178(7):721–728. 10.1164/rccm.200803-436OC 18596224 PMC2556454

[76] Latzin P Roosli M Huss A: Air pollution during pregnancy and lung function in newborns: a birth cohort study. *Eur Respir J.* 2009;33(3):594–603. 10.1183/09031936.00084008 19010988

[77] Hafström O Milerad J Sundell HW: Altered Breathing Pattern after Prenatal Nicotine Exposure in the Young Lamb. *Am J Respir Crit Care Med.* 2002;166(1):92–97. 10.1164/rccm.2107082 12091177

[78] Hayatbakhsh MR Sadasivam S Mamun AA: Maternal smoking during and after pregnancy and lung function in early adulthood: a prospective study. *Thorax.* 2009;64(9):810–814. 10.1136/thx.2009.116301 19525264

[79] GBD 2015 Tobacco Collaborators, Reitsma MB Fullman N: Smoking prevalence and attributable disease burden in 195 countries and territories, 1990-2015: a systematic analysis from the Global Burden of Disease Study 2015. *Lancet.* 2017;389(10082):1885–1906. 10.1016/S0140-6736(17)30819-X 28390697 PMC5439023

[80] Lange S Probst C Rehm J: National, regional, and global prevalence of smoking during pregnancy in the general population: a systematic review and meta-analysis. *Lancet Glob Heal.* 2018;6(7):e769–e776. 10.1016/S2214-109X(18)30223-7 29859815

[81] Upton MN Smith GD McConnachie A: Maternal and Personal Cigarette Smoking Synergize to Increase Airflow Limitation in Adults. *Am J Respir Crit Care Med.* 2004;169(4):479–487. 10.1164/rccm.200211-1357OC 14630616

[82] Hu LW Yang M Chen S: Effects of in utero and Postnatal Exposure to Secondhand Smoke on Lung Function by Gender and Asthma Status: The Seven Northeastern Cities (SNEC) Study. *Respiration.* 2017;93(3):189–197. 10.1159/000455140 28092910

[83] Tsai HJ Liu X Mestan K: Maternal cigarette smoking, metabolic gene polymorphisms, and preterm delivery: new insights on GxE interactions and pathogenic pathways. *Hum Genet.* 2008;123(4):359–69. 10.1007/s00439-008-0485-9 18320229 PMC2852624

[84] Le Souëf PN: Pediatric origins of adult lung diseases. 4. Tobacco related lung diseases begin in childhood. *Thorax.* 2000;55(12):1063–7. 10.1136/thorax.55.12.1063 11083894 PMC1745663

[85] Guerrant RL DeBoer MD Moore SR: The impoverished gut—a triple burden of diarrhoea, stunting and chronic disease. *Nat Rev Gastroenterol Hepatol.* 2013;10(4):220–229. 10.1038/nrgastro.2012.239 23229327 PMC3617052

[86] Humphrey JH: Child undernutrition, tropical enteropathy, toilets, and handwashing. *Lancet.* 2009;374(9694):1032–1035 . 10.1016/S0140-6736(09)60950-8 19766883

[87] Kelly P Menzies I Crane R: Responses of small intestinal architecture and function over time to environmental factors in a tropical population. *Am J Trop Med Hyg.* 2004;70(4):412–9 . 10.4269/ajtmh.2004.70.412 15100456

[88] Chunxi L Haiyue L Yanxia L: The Gut Microbiota and Respiratory Diseases: New Evidence. *J Immunol Res.* 2020;2020:2340670. 10.1155/2020/2340670 32802893 PMC7415116

[89] Schwarzer M Kassem M Storelli G: *Lactobacillus plantarum* strain maintains growth of infant mice during chronic undernutrition. *Science.* 2016;351(6275):854–7. 10.1126/science.aad8588 26912894

[90] Blanton LV Charbonneau MR Salih T: Gut bacteria that prevent growth impairments transmitted by microbiota from malnourished children. *Science.* 2016;351(6275). 10.1126/science.aad3311 26912898 PMC4787260

[91] Charbonneau MR O'Donnell D Blanton LV: Sialylated Milk Oligosaccharides Promote Microbiota-Dependent Growth in Models of Infant Undernutrition. *Cell.* 2016;164(5):859–71 . 10.1016/j.cell.2016.01.024 26898329 PMC4793393

[93] Ranucci G Buccigrossi V deFreitas MB: Early-Life Intestine Microbiota and Lung Health in Children. *J Immunol Res.* 2017;2017:8450496. 10.1155/2017/8450496 29359170 PMC5735664

[92] Budden KF Gellatly SL Wood DLA: Emerging pathogenic links between microbiota and the gut-lung axis. *Nat Rev Microbiol.* 2017;15(1):55–63. 10.1038/nrmicro.2016.142 27694885

[94] Azad MB Kozyrskyj AL: Perinatal Programming of Asthma: The Role of Gut Microbiota. *Clin Dev Immunol.* 2012;2012:932072. 10.1155/2012/932072 22110540 PMC3216351

[95] Tai A: Association between childhood asthma and adult chronic obstructive pulmonary disease. *Minerva Pneumol.* 2017;56:134–138. 10.23736/S0026-4954.17.01780-1 24646659

[96] Oswald H Phelan PD Lanigan A: Childhood asthma and lung function in mid-adult life. *Pediatr Pulmonol.* 1997;23(1):14–20. 10.1002/(sici)1099-0496(199701)23:1<14::aid-ppul2>3.0.co;2-p 9035194

[97] Naylor C Lu M Haque R: Environmental Enteropathy, Oral Vaccine Failure and Growth Faltering in Infants in Bangladesh. *EBioMedicine.* 2015;2(11):1759–66. 10.1016/j.ebiom.2015.09.036 26870801 PMC4740306

[98] Priemé H Loft S Klarlund M: Effect of smoking cessation on oxidative DNA modification estimated by 8-oxo-7,8-dihydro-2’-deoxyguanosine excretion. *Carcinogenesis.* 1998;19(2):347–351. 10.1093/carcin/19.2.347 9498287

[99] Levels and trends in child malnutrition. UNICEF / WHO / World Bank Group Joint Child Malnutrition Estimates.2019 Reference Source

[100] Minelli C Dean CH Hind M: Association of Forced Vital Capacity with the Developmental Gene NCOR2. *PLoS One.* 2016;11(2):e0147388. 10.1371/journal.pone.0147388 26836265 PMC4737618

[101] Jackson VE Latourelle JC Wain LV: Meta-analysis of exome array data identifies six novel genetic loci for lung function. *Wellcome Open Res.* 2018;3:4. 10.12688/wellcomeopenres.12583.3 30175238 PMC6081985

[102] Loth DW Artigas MS Gharib SA: Genome-wide association analysis identifies six new loci associated with forced vital capacity. *Nat Genet.* 2014;46(7):669–677. 10.1038/ng.3011 24929828 PMC4140093

[103] Soler Artigas M Loth DW Wain LV: Genome-wide association and large-scale follow up identifies 16 new loci influencing lung function. *Nat Genet.* 2011;43(11):1082–90. 10.1038/ng.941 21946350 PMC3267376

[104] Repapi E Sayers I Wain LV: Genome-wide association study identifies five loci associated with lung function. *Nat Genet.* 2010;42(1):36–44. 10.1038/ng.501 20010834 PMC2862965

[105] Miller S Melén E Merid SK: Genes associated with polymorphic variants predicting lung function are differentially expressed during human lung development. *Respir Res.* 2016;17(1):95. 10.1186/s12931-016-0410-z 27473260 PMC4966770

[106] Chu AY Workalemahu T Paynter NP: Novel locus including FGF21 is associated with dietary macronutrient intake. *Hum Mol Genet.* 2013;22(9):1895–902. 10.1093/hmg/ddt032 23372041 PMC3612009

[107] Zhao J Bradfield JP Zhang H: Role of BMI-Associated Loci Identified in GWAS Meta-Analyses in the Context of Common Childhood Obesity in European Americans. *Obesity (Silver Spring).* 2011;19(12):2436–2439. 10.1038/oby.2011.237 21779088

[108] Perignon M Fiorentino M Kuong K: Stunting, poor iron status and parasite infection are significant risk factors for lower cognitive performance in Cambodian school-aged children. *PLoS One.* 2014;9(11):e112605. 10.1371/journal.pone.0112605 25405764 PMC4236074

[109] Barton SJ Murray R Lillycrop KA: FUT2 Genetic Variants and Reported Respiratory and Gastrointestinal Illnesses During Infancy. *J Infect Dis.* 2019;219(5):836–843. 10.1093/infdis/jiy582 30376117 PMC6687504

[110] Mishra N: Review Data ( GBD).csv. *figshare.*Dataset.2020 10.6084/m9.figshare.12278129.v1

