# Supplementary material for: Low lung function in the developing world is analogous to stunting: a review of the evidence
Source: Wellcome Open Res. 2020 Nov 18;5:147. Originally published 2020 Jun 24. [Version 2] doi: 10.12688/wellcomeopenres.15929.2 (PMC7745193; doi:10.12688/wellcomeopenres.15929.2)
Supplement: Peer review history

# Peer review: Reviewer response for version 2

**DOI:** 10.21956/wellcomeopenres.18087.r41488

**Published:** 2020-12-16

**Authors:** Ashutosh Nath Aggarwal

**Affiliations:** 1 Department of Pulmonary Medicine, Postgraduate Institute of Medical Education and Research (PGIMER), Chandigarh, Punjab and Haryana, India

This revision has tried to address most of my concerns, and is much improved in both content and direction. I consider some residual differences in opinion to be an essential part of the scientific thought process which should not lessen the merit of the current manuscript. I have no additional comments to offer.

Is the work clearly and accurately presented and does it cite the current literature?

Partly

If applicable, is the statistical analysis and its interpretation appropriate?

Partly

Are all the source data underlying the results available to ensure full reproducibility?

Yes

Is the study design appropriate and is the work technically sound?

Partly

Are the conclusions drawn adequately supported by the results?

Partly

Are sufficient details of methods and analysis provided to allow replication by others?

Partly

Reviewer Expertise:

Epidemiology of chronic respiratory disorders, Pulmonary function testing, health outcomes research

I confirm that I have read this submission and believe that I have an appropriate level of expertise to confirm that it is of an acceptable scientific standard.

# Peer review: Reviewer response for version 1

**DOI:** 10.21956/wellcomeopenres.17471.r39560

**Published:** 2020-08-03

**Authors:** Ashutosh Nath Aggarwal

**Affiliations:** 1 Department of Pulmonary Medicine, Postgraduate Institute of Medical Education and Research (PGIMER), Chandigarh, Punjab and Haryana, India

**General:**

- The manuscript attempts to visualize the differences in lung function across various ethnic groups from a different perspective, one that is slowly being recognized as perhaps being of some importance. However, the overall handling of the issue and its presentation leave much to be desired. In their zeal to increase the width of discussion (and trying to evaluate variables that may or may not be even remotely connected with the problem at hand), the authors compromise a lot on the depth of their discussion. The manuscript essentially tries to identify factors associated with perinatal and childhood stunting (and related conditions), on which extensive literature is available. The manuscript also explores literature (to a limited extent) to try and identify if some of these factors also correlate with diminished pulmonary function, and tries to build a viewpoint that the two appear analogous. I personally am not comfortable with this translation from ‘association’ to ‘analogy’. For instance, tobacco smoking is known to be associated with several developmental, inflammatory, infective, metabolic, degenerative and malignant disorders, but one cannot conclude that all these hundreds of health conditions are analogous to each other just because they share one risk factor, one among the several that is associated with each clinical scenario. In addition, the entire presentation attempts to build a concept of causality, and we all understand the perils of extrapolating associations to causality.
- The manuscript, although being close to a systematic review (as claimed by authors), presents only one side of the viewpoint, a viewpoint that authors have probably pre-conceived to be correct. I cannot figure out how during their review of evidence they did not come across or discuss even a single study that is contrary to this viewpoint. Although I partly agree with this stated viewpoint, but even as a lay person I can raise several objections. For instance, women across the globe have poorer lung function than men. Does that imply that all women are ‘stunted’ (based on their lower height, weight, chest circumference, etc.) and hence exhibit a lower vital capacity? In India, north Indians are generally taller and less stocky than south Indians and show better lung function. Would it mean that south Indians are uniformly less privileged, and hence ‘more stunted’, than north Indians in this country? It seems too simplistic and naïve to conclude that poor lung function in certain populations is almost completely related to poor lung growth, which in turn is almost completely related to malnutrition, poor socio-economic status, etc. For instance, a study from the UK concluded that chest dimensions did not explain the substantial effect of ethnicity (whites vs. Asians) on pulmonary function in children aged 6-11 years (Whittaker *et al.*, 2005 ^1^). Similarly, a study on college students aged 18-23 years in the UK recruited 112 subjects of two ethnicities with similar parental socio-economic environment and birth weight, yet their difference in FVC was 0.81 L after adjustment for gender, age, anthropometry and social variables (Saad *et al.*, 2017 ^2^). There are more such studies that seem to fulfil the authors’ eligibility criteria but are neither cited nor discussed. I don’t feel that it is so simple (as the authors’ make it out to be) to untangle the influences from deprivation and genetics on pulmonary function.

 

**Abstract:**

- The abstract is a well-written summary but seems totally disconnected to the manuscript. The background here is quite different from what is presented as the Introduction in the main text. The methods too are different with respect to databases and datasets queried for literature search. There is no mention of following PRISMA guidelines in the manuscript.

 

**Introduction:**

- The authors state certain facts with certainty and draw conclusions right away. For instance, … "both lung function and early growth are strongly associated with and defined by linear growth, influenced by similar perinatal factors, and culminate in an elevated risk of non-communicable diseases sharing chronic inflammatory origins" …, and … "analogous nature of restrictive lung function and stunting would imply" …, etc. I believe these are actually the research questions here that they are trying to explore. A proper justification and statement regarding need for this research would be welcome.

 

**Methods:**

- A proper search strategy is lacking, and the keywords provided are too general. I am sure any bibliographic search would have returned tonnes of citations, and I am not sure how the authors sifted through for relevant publications. I am also not sure how the search terms stated in table 1 could have been used to identify relevant articles, since these appear too restrictive in the single phrases that they employ. Yet a large body of evidence is provided. For instance, the genetic studies listed in table 2 could not have been derived from the search string “stunting AND low lung function AND genetic NOT animal NOT cystic fibrosis”. Studies with unclear methodology were excluded, but there is no explanation how this was determined. Overall, the section is nowhere close to what PRISMA guidelines advocate.

 

**Results:**

- A major drawback of the manuscript is that it tries to fit a narrative review in an original article format. This may have been possible if this were a systematic review, but clearly that is not the case. So the Results section is basically a summary of some articles considered important by the authors, expression of authors’ perceptions about the evidence, and conclusions on potential pathways even without sufficient evidence. There is no Discussion section. A lot of statements do not actually stem from published data reported by the authors, but are rather thoughts or hypotheses. Overall, this seems straight out of an informal literature review for a thesis/dissertation, rather than the Results section of an original research article. I would expect slightly better focus, and a better review of strength and weakness of data, before jumping to interpretation.
- Some studies are not referenced in the bibliography (Todisco et al on page 6, column 1).
- The section on impoverished gut seems totally out of context in this article as it does not provide any evidence correlating abnormalities in gut microbiota to poor lung function. Asthmatic phenotype cannot be equated with poor lung function.
- In the section on genetic modulators I am not clear how the conclusion, that genes common to both FVC and stunting were associated with early development, morphogenesis and malnutrition, was arrived at.

 

**Conclusions:**

- At this stage, the conclusions seem largely conjectural and not completely borne out by data presented (and not presented). Most of this section seems superfluous and largely irrelevant to the present discussion. It should be crisp and to the point.

Is the work clearly and accurately presented and does it cite the current literature?

Partly

If applicable, is the statistical analysis and its interpretation appropriate?

Partly

Are all the source data underlying the results available to ensure full reproducibility?

Yes

Is the study design appropriate and is the work technically sound?

Partly

Are the conclusions drawn adequately supported by the results?

Partly

Are sufficient details of methods and analysis provided to allow replication by others?

Partly

Reviewer Expertise:

Epidemiology of chronic respiratory disorders, Pulmonary function testing, health outcomes research

I confirm that I have read this submission and believe that I have an appropriate level of expertise to confirm that it is of an acceptable scientific standard, however I have significant reservations, as outlined above.

## References

[1] : Are ethnic differences in lung function explained by chest size?. *Arch Dis Child Fetal Neonatal Ed*.2005;90(5) : 10.1136/adc.2004.062497 F423-8 10.1136/adc.2004.062497 15871993PMC1721951

[2] : Explaining ethnic disparities in lung function among young adults: A pilot investigation. *PLoS One*.2017;12(6) : 10.1371/journal.pone.0178962 e0178962 10.1371/journal.pone.0178962 28575113PMC5456386

# Response

**Published:** 2020-11-07

**Authors:** Anurag Agrawal

**Affiliations:** CSIR Institute of Genomics and Integrative Biology, India

1. “Issue - translation from ‘association’ to ‘analogy’” **Main Response – Explanation for describing this as an analogy has been added. The abstract has been modified slightly to represent the content of the manuscript better. **
2. “The manuscript, although being close to a systematic review (as claimed by authors), presents only one side of the viewpoint” - **A detailed explanation for why we could not conduct a systematic review on the subject has been presented both in the abstract and listed as a limitation. Attempts for a balanced literature review were made, other references were added.**
3. It seems too simplistic and naïve to conclude that poor lung function in certain populations is almost completely related to poor lung growth, which in turn is almost completely related to malnutrition, poor socio-economic status, etc. , impossible to untangle the influences from deprivation and genetics on pulmonary function. **Response: There are points in the manuscript where differences between the nature of both conditions were described - these differences have been explained in greater detail. The role of genetics has also been described in greater detail. Both studies and others similar to them have been included in the ‘interplay of ethnicity and access’ section of the manuscript.**
4. Abstract: The background here is quite different from what is presented as the Introduction in the main text. The methods too are different with respect to databases and datasets queried for literature search. There is no mention of following PRISMA guidelines in the manuscript. **Response: The abstract has been modified to reflect the contents of the manuscript better. PRISMA guidelines have been followed to the extent possible and reported in the methods section.**
5. Authors state certain facts with certainty and draw conclusions right away. A proper justification and statement regarding the need for this research would be welcome.
**Any conclusive statements about the hypothesis have been removed from the beginning of the manuscript.**
6. A proper search strategy is lacking, and the keywords provided are too general.. Overall, the section is nowhere close to what PRISMA guidelines advocate. **Response: Methods re-written, results modified accordingly, new references added**
7. A major drawback of the manuscript is that it tries to fit a narrative review in an original article format. There is no Discussion section. A lot of statements do not actually stem from published data reported by the authors, but are rather thoughts or hypotheses.

**Response** **:** **Additional references provided.**

- Some studies are not referenced in the bibliography (Todisco et al on page 6, column 1).

**Response** **:** **Additional references provided.**

- The section on impoverished gut seems totally out of context in this article as it does not provide any evidence correlating abnormalities in gut microbiota to poor lung function.

**Response** **:** **Additional references provided.**

- Asthmatic phenotype cannot be equated with poor lung function. – **Correction made.**

In the section on genetic modulators I am not clear how the conclusion, that genes common to both FVC and stunting were associated with early development, morphogenesis and malnutrition, was arrived at. **Response: Additional explanation provided.**

# Peer review: Reviewer response for version 1

**DOI:** 10.21956/wellcomeopenres.17471.r39246

**Published:** 2020-07-10

**Authors:** Vishal Bansal

**Affiliations:** 1 Department of Physiology, Vallabhbhai Patel Chest Institute, New Delhi, Delhi, India

The authors have touched upon a clinically relevant topic i.e. impact of stunting on lung function in developing world.

 They have critically reviewed the available literature in a crisp and appropriate manner.

 Though they have focused mainly on IUGR and malnutrition with brief mention of obesity; it would add to value of the manuscript if more literature is added on the correlation between obesity and stunting.

Is the work clearly and accurately presented and does it cite the current literature?

Yes

If applicable, is the statistical analysis and its interpretation appropriate?

Not applicable

Are all the source data underlying the results available to ensure full reproducibility?

Yes

Is the study design appropriate and is the work technically sound?

Yes

Are the conclusions drawn adequately supported by the results?

Yes

Are sufficient details of methods and analysis provided to allow replication by others?

Yes

Reviewer Expertise:

Cardio-respiratory physiology, Cardio-pulmonary rehabilitation.

I confirm that I have read this submission and believe that I have an appropriate level of expertise to confirm that it is of an acceptable scientific standard.

## References

[1] : Overweight, stunting, and concurrent overweight and stunting observed over 3 years in Vietnamese children. *Glob Health Action*.2018;11(1) : 10.1080/16549716.2018.1517932 1517932 10.1080/16549716.2018.1517932 30253704PMC6161605
